# Benzol in Leukæmia

**Published:** 1914-12-26

**Authors:** 


### BENZOL IN LEUKÆMIA.

A few years ago the stereotyped treatment of all forms of leukæmia was the administration of arsenic, usually in the form of cacodylate of soda. This treatment was almost always unavailing, though occasional temporary improvement seemed to follow its use. Then *x*-ray treatment came into fashion: directed, in the spleno-medullary form of the disease, to the spleen and the long bones, and in the lymphatic forms to the bones alone. Accumulated experience proves that it is only now and then that any great benefit arises, and certainly it is impossible to describe it in any sense as a cure. A year or two ago the administration of benzol was advocated for leukæmia, but the reports of different observers are not by any means unanimous. It is, however, the conviction of many physicians that the prospect of benefit from benzol is greater than that from any other known method.

One of the important points to bear in mind is that the beneficial effect lasts for some considerable time after the course of benzol has terminated. It is in consequence not wise to carry on the treatment until the number of white corpuscles has been reduced absolutely to normal; it is better to discontinue it before such a stage is reached, as the drop will progress after the benzol has been stopped. Another important caution is that benzol produces gastro-intestinal irritation, evidenced by nausea, vomiting, flatulence, and pain. Dizziness, albuminuria, bronchial irritation, and hæmorrhages from mucous membranes may also occur. Individual tolerance of benzol varies. A good plan is to start with ten minims of benzol and ten of olive oil, given together in a gelatine capsule: one such capsule is given after meals three times a day at first, and the dose can be increased to six or even ten a day.

One of the most recent pronouncements upon this treatment is that of Levison*; it reflects fairly accurately what the majority of other observers have found, and is therefore worth quotation. Benzol, he says, is a symptomatic remedy of great value in leukæmia; and the action is not restricted to any one type of leukæmia, but applies to all. When it cannot be tolerated by the mouth, it may be tried subcutaneously or by the rectum. Benzol causes at first an increase in the white blood-cells, followed by a marked fall. This drop may go below normal, even to a complete absence of white cells if the use of benzol be unduly prolonged. The administration should therefore always be stopped before the white cells reach a normal figure. In moderate doses benzol has also a favourable action upon the red blood-cells and upon their hæmoglobin. Whenever possible the use of *x*-rays should be combined with that of benzol. The drug has also a favourable action to a limited degree on some types of pseudo-leukæmia. Levison possibly includes in this that rare disease chloroma; other observers have also reported that chloroma is sometimes amenable to benzol.

* *Interstate Medical Journal*, July 1914.

